# Sorbent Film-Coated Passive Samplers for Explosives Vapour Detection Part B: Deployment in Semi-Operational Environments and Alternative Applications

**DOI:** 10.1038/s41598-018-24245-x

**Published:** 2018-04-11

**Authors:** Gillian L. McEneff, Alexandra Richardson, Tony Webb, Dan Wood, Bronagh Murphy, Rachel Irlam, Jim Mills, David Green, Leon P. Barron

**Affiliations:** 1 King’s Forensics, School of Population Health & Environmental Sciences, Faculty of Life Sciences & Medicine, King’s College London, SE1 9NH London, United Kingdom; 2 Threat Mitigation Technologies, Metropolitan Police Service, 113 Grove Park, London, SE5 8LE United Kingdom; 3 Air Monitors Ltd., 2/3 Miller Court, Severn Drive, Tewkesbury, Gloucestershire GL20 8DN United Kingdom

## Abstract

The application of new sorbent-film coated passive samplers for capture of bulk commercial and military explosives vapours in operationally relevant spaces such as luggage, rooms, vehicles and shipping containers is presented. Samplers were easily integrated with in-service detection technologies with little/no sample preparation required. Ethylene glycol dinitrate (EGDN) was detected within 4 h in a container holding a suitcase packed with 0.2 kg Perunit 28E. Within a 22,000 dm^3^ room, 1 kg of concealed Seguridad was detected within 24 h and in an adjoining room within 7 days. Exposed samplers also successfully captured components of 1 kg TNT after 72 h and 1 kg concealed Perunit 28E after 6 h in both a furnished room and a large, partially filled shipping container. For the latter, samplers captured detectable residues outside the container after 24 h and were stable during wet weather for 72 h. A one-week trial at three operationally relevant venues including a university, a theatre and a government building revealed a nuisance positive rate of <1.4% (n = 72). Finally, two alternative applications are presented for extraction of liquid samples and use a particulate contact swab showing flexibility for a range of different search activities.

## Introduction

Methods available for sampling and concentration of explosives vapours are mostly based on active technology where a pump is used to draw air either directly into the detector or via a tube, through or over collection filters or packed sorbent beds. Detection is then normally performed via thermal desorption by mass spectrometry (TD-MS) or ion mobility spectrometry (IMS) and analysis is achieved in a matter of seconds^1^. The latter technology in particular remains one of the most widely used methods for screening explosive threats at airports^2^. Canine olfaction is also an efficient approach used by both police and the military to offer a quick, flexible and mobile method for the identification of concealed vapour sources. However, dogs are unable to identify the type or quantity of explosive detected and are influenced by various factors that can affect their reliability including the training received, handler’s ability, length of shift, age, health and distractions in the immediate vicinty^3,4^. For vapour sampling during venue searches, hand-held detection systems are often used, but trained operators are required to collect samples, making it relatively labour intensive, costly and time consuming for repetitive searching of large areas. There have also been issues with the level of false positives measured, making them difficult to use for rapid venue searches^5^. There is still a need for a continuous, time-integrated, autonomous and low-cost explosive vapour monitoring system to further enhance the assurance of existing operational screening technologies for the detection of more volatile explosive components.

With the demand to detect a larger range of explosives at lower concentrations, more selective and sensitive explosive vapour sensors have been recently developed, however, few technologies have successfully transitioned to field use^6^. Colorimetric and fluorometric sensor arrays, also known as optoelectronic noses, have been shown to be a rapid and sensitive approach for field detection of explosive vapours, such as 2,4-DNT for buried TNT in soils^7^, nitro-organic compounds and peroxides^8,9^. As described by Lefferts and Castell (2015), polymers, nanomaterials, cantilevers and electronic noses all have the capability for explosive vapour detection but when used in combination, the added functionality offered could provide heightened sensitivity and confirmation of compound identity^6^. Molecularly imprinted polymers have been developed for the detection of TNT vapours, but though these polymers offer high analyte selectivity, they suffer operationally where complex matrices can hinder or block analyte uptake within the imprinted cavities^10^. Using a combination of molecular imprinting and charge transfer complex luminescence, an explosive detection sensor was developed that can be deployed as a hand-held vapour sniffer, a vapour imaging device for unexploded ordinance and land mines, and with minor variations, an explosives detection device in water^11^. Few existing devices can be used for both particulate and vapour detection yet reliable explosive identification in solid, gas and liquid phases is desirable.

Where highly sensitive and selective methodologies are developed, the potential for false or nuisance positives arguably rises. While detection of an analyte may be confirmed, no imminent viable threat is often found, *i.e*. no explosive device. Therefore, understanding the occurrence of compounds, and their quantities, is critical to implementation of such sensitive devices for routine operation. In the UK and the USA, large-scale studies have been carried out to measure the occurrence of high-order explosives in public locations, including transport sites, and at public areas with higher human contact such as park benches and public telephones^12–14^. In the more recently published UK study in 2004, particulate sampling (swabs wetted with 1:1 ethanol:water and vacuuming) was not only performed at transport sites around four major UK cities (Birmingham, Cardiff, Glasgow and Manchester), but also at hotels, private houses, vehicles and on clothing. Analysis was performed using gas chromatography-thermal energy analysis detection (GC-TEA), GC coupled to mass spectrometry (GC-MS) and liquid chromatography-MS (LC-MS). Results from all three of these large-scale studies showed that selected organic explosives were uncommon in most public places, and in the case of the most recent UK study, only 4 of 501 samples yielding positive results at very low concentrations i.e. 7.5 ng of cyclotrimethylene-trinitramine (RDX), 3.6 ng of nitroglycerin (NG) and 15.2 ng of 2,4-dinitrotoluene (2,4-DNT)^13^. Explosives such as TNT, RDX and octahydro-1,3,5,7-tetranitro-1,3,5,7-triazine (HMX) have been previously found in effluents from explosive manufacturing sites as well as surface water and soil samples, due to dissemination into the environment post-military disposal operations^15–17^. More recently, 2,4-DNT and 2,6-DNT have been detected in raw sewage collected from municipal wastewater treatment plants located in the United Kingdom and Finland, respectively, at concentrations ranging from mid ng L^−1^ to low µg L^−1^ levels^18,19^. Clearly, based on some of these previous occurrence studies, the potential for vapours to exist is plausible for some of the more volatile compounds such as DNT or TNT. Very little information exists on airborne/vapour occurrence and this remains a challenge for search teams to set robust thresholds to minimise false or nuisance positives.

The aim of this work was to assess the performance of a new passive air sampler (also see Part A of this work^20^) in various simulated and operational environments typically encountered by defensive search teams. In addition, its applicability for explosives sampling in the liquid phase and as a particulate swab was investigated. The use of a variety of commercial and military explosives to test sampler performance provides new knowledge on the kinetics of explosive vapour dissipation in operationally relevant and well-defined spaces as well as robust establishment of best practice guidelines in terms of sampler location, effective distance, exposure times, and the rate of false/negative alarms.

## Results and Discussion

### Rationale for trials

Several trials were conducted using bulk explosives to assess the performance of the sampler for trapping vapours and also their usefulness in alternative applications. Unfortunately, peroxide explosives testing was not permitted at these trial sites and were not investigated beyond laboratory-scale experiments in Part A of this work. However, all other compounds could potentially be detected in trials using these commercial and military grade explosives, either as the intact explosives vapours themselves (TNT) or via associated transformation products (e.g. DNTs etc.). All 15 explosives were monitored, including peroxides, even if the deployed explosive did not contain that marker. Several variables were tested in this assessment including (a) void type, (b) void size, (c) concealment, (d) sampler distance from source, (e) time from explosive deployment to detection, (f) false/nuisance positives and (g) alternative applications. Across Trials 1–5, complexity generally increased mainly in terms of the degree of concealment and the void size. In all trials, focus was placed on the typical types of operational environments frequently searched by police. Earlier trials used the bare coated samplers and later trials incorporated an optimised discrete housing for mounting and devices were retested in voids which both included and excluded the presence of explosive sources to assess false negatives or false/nuisance positives, respectively. No trial included a ‘pre-soak’ period for the explosives source(s) before deployment of passive samplers. The ultimate goal was to characterise the worst-case scenario to include the dissipation time for a freshly deployed, concealed explosive material in a large void (as presented in Trials 3/4).

### Trial 1: Preliminary trials in baggage and vehicles

All preliminary trials involved the exposure of the bare coated sampler to single explosive sources in smaller void volumes with reduced interferences (sealed vents and no cargo) over a relatively short period of time (up to 22/47 h). For the vehicle exposure, samplers were exposed at one fixed location from an unconcealed source (see Table 1 for further details).

**Table 1:** Detection of commercial and military blasting explosives in preliminary exposures in Trial 1 using suitcases and vehicles and analysed with LC-UV, TD-MS and IMS.

Void type (volume)	Sampler location	Explosive type (weight)	Exposure time (h)	Analyte detection method and time		
				*LC-UV*	*TDMS*	*IMS*
Suitcase (20 dm^3^) in a plastic container (64 dm^3^)	Outside of suitcase, but inside container box (n = 24)	Perunit 28E (0.2 kg)	47	EGDN ≥ 4 h	EGDN ≥ 4 h	EGDN ≥ 23 h
		TNT (0.6 kg)	47	n.d.	n.d.	n.d.
		Semtex 1 H (3 kg)	47	n.d.	n.d.	n.d.
Vehicle (9000 dm^3^ Transit Van)	At rear door, approx. 4 m from source (n = 12 for 22/23 h exposures and n = 24 for 47 h exposure)	Perunit 28E (0.4 kg)	23	EGDN ≥ 4 h	EGDN ≥ 4 h	EGDN ≥ 4 h
		Perunit 28E (4 kg)	22	EGDN ≥ 4 h NG ≥ 22 h	EGDN ≥ 4 h NG ≥ 22 h	EGDN ≥ 4 h NG ≥ 22 h
		TNT (3 kg)	47	n.d.	DNT ≥ 23 h	TNT ≥ 23 h

Results for preliminary trial experiments and sampler integration with LC-UV, IMS and TD-MS analysis are summarised in Table 1. EGDN was detected both on samplers collected from inside and outside of the suitcase after 4 h of exposure to Perunit 28E. The detection of such similar signal intensities for EGDN after 47 h suggests equilibrium was reached, even with complete concealment within wrapped cotton towels inside each of the cases. Despite low vapour pressures for components in Semtex 1 H, the additional presence of 2,3-dimethyl-2,3-dinitrobutane (DMNB) as a marking agent often aids detection via its vapour. However, DMNB was not detected on any of the samplers collected over 47 h. TNT was not detected during the suitcase trial. Samplers collected from the control suitcase and the tent showed low levels of EGDN highlighting the transport of EGDN vapours through various materials. For vehicle-scale exposures, EGDN was again detected after 4 h for both 0.4 and 4 kg Perunit 28E sources, and Ng was also detected after 22 h exposure to the 4 kg Perunit 28E source only. TNT and DNT were detected using IMS and TDMS analysis, respectively, after 23 h exposure to a TNT-based explosive. No concealment was performed internally within the vehicle and these results agreed with the laboratory-scale exposures in Part A. Although explosives were not concealed internally, EGDN was unexpectantly detected outside of the vehicle holding 4 kg of Perunit 28E using IMS using a Morpho Detection MobileTrace detector (Smiths Detection, London, UK), but only at specific areas; under the van (confirmed by samplers analysed by LC-UV and TDMS), at the back door (confirmed by samplers analysed by IMS and TDMS) and side of the van (confirmed by samplers analysed using LC-UV, IMS and TDMS). All other areas tested negative with IMS, suggesting EGDN detection came from within the van, as opposed to the background site. It was unclear whether an explosive substance concealed inside a vehicle would have yielded the same result.

### Trial 2: Room-scale exposures and determination of optimal sampler range

In this trial, unhoused film-coated samplers were placed at several heights and distances from a single explosive source concealed within a small locked cabinet in a room measuring more than double the previous vehicle exposure void. Samplers were placed at various locations within the voids (with minimised physical/chemical interferences, i.e. sealed vents and no contents) and externally in surrounding rooms for a longer exposure period of up to 7 days (see experimental for further details).

**Figure 1:**
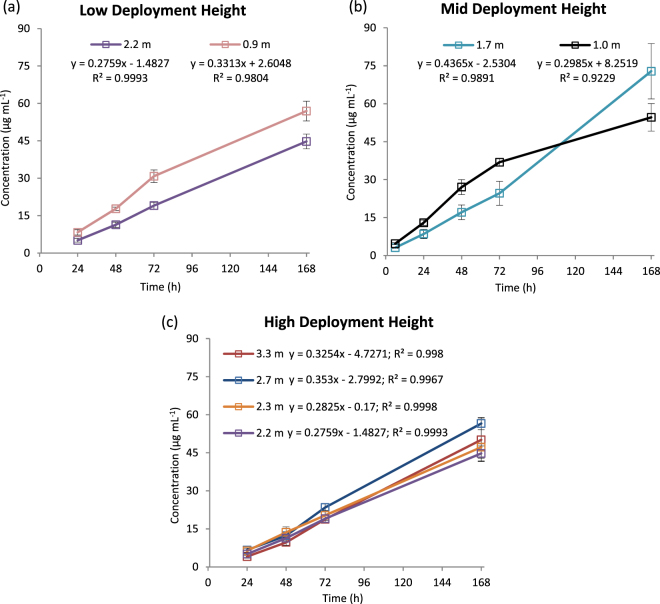
Room-scale uptake of EGDN over 168 h on samplers located at (**a**) low, (**b**) mid and (**c**) high heights in Room A and at varying distances from the source in Trial 2.

Figure 1 shows that EGDN was detected from 6 h on passive samplers located at mid-height and positioned above the source in Room A. After 24 h, EGDN was detected on all samplers at the extremities of Room A and at all heights. In terms of best location for sampler placement within a room, all heights and distances measured in Room A showed similar uptake of EGDN from 24 h and therefore flexibility exists, though this may depend on the explosive. However, it is recommended that samplers are exposed for at least this period (based on an approximate 22,000 dm^3^ room volume). For high vapour pressure compounds and for those with average densities lower than air, location of samplers above reach is recommended to avoid tampering. However, samplers may also be placed lower down and out of sight (e.g. underneath furniture or other fixtures). This may be important in the case of TATP vapour, for example, which has been shown to sink when released in stable, ambient air in a double T-shaped chamber, with highest concentrations measured below the sample source^21^. It is recommended that at least duplicate samplers are deployed at each location within a room for added assurance. If a larger room is used, multiple sets of samplers should be deployed to ensure the widest coverage of the monitored space in the same 24-h timeframe. For the adjoining Room B, EGDN was first detected on all samplers after 168 h exposure, which included the time for dissipation through a door which was kept closed (door frame not sealed) and only opened briefly during sampler collection times (see Figure S1 for room set-up). Similar to the negative controls in Room A, partially resolved interferent peaks from EGDN were apparent using LC-UV analysis. Uptake may have occurred sooner than the reported times, but was either obscured by this interfering peak or was present at <LOD for LC-UV detection. It is important to also note that following initial detection of EGDN on samplers in Room A (between 6 and 24 h), no false negative results occurred thereafter, despite the analysts entering/exiting the room for collection of samplers only at the specific time points.

**Figure 2:**
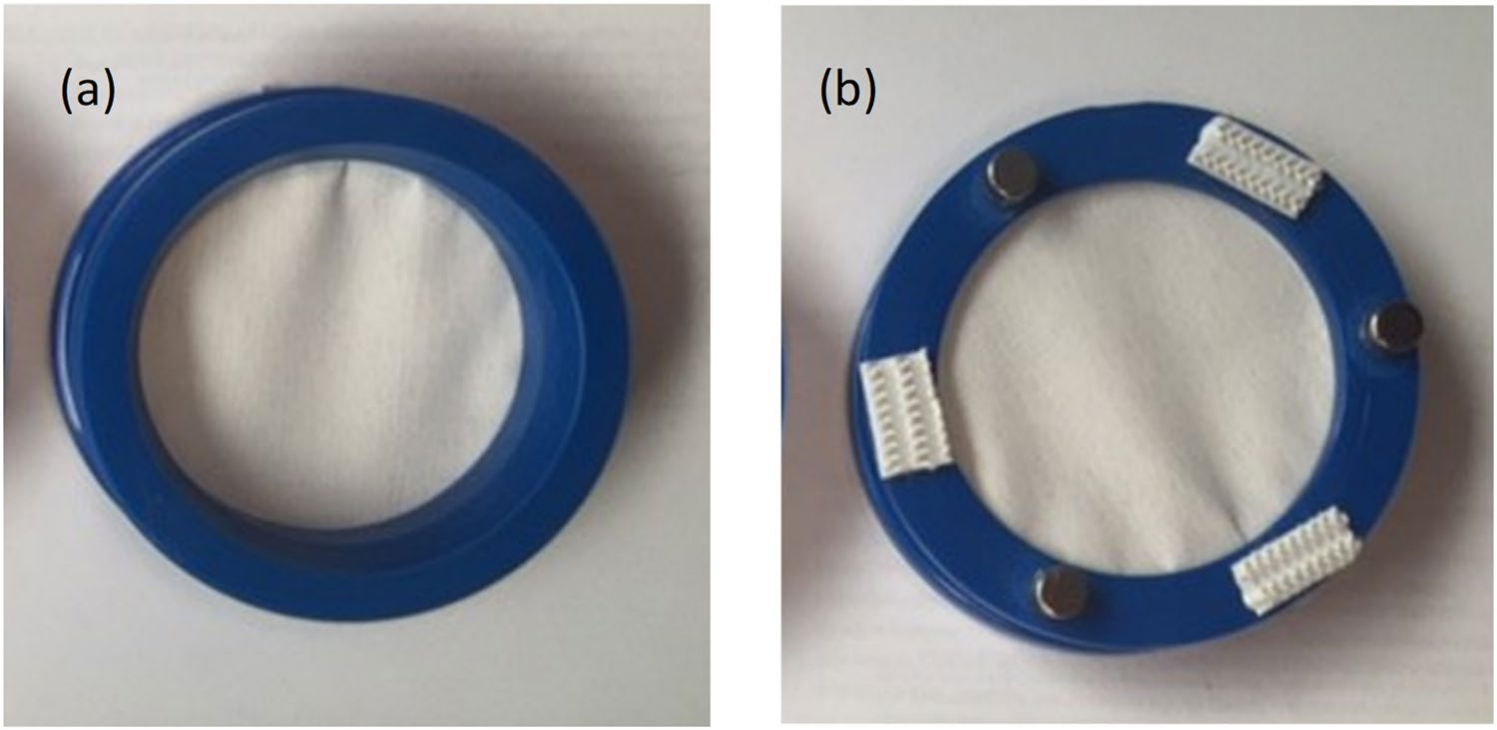
The final housed passive sampler design (**a**) front and (**b**) back with attachments shown i.e. magnets and hook and loop (Velcro) fasteners.

During this trial, a small number of different sampler housing designs were tested to measure the impact on performance. Uptake of EGDN was highest without any housing and in comparison, uptake on housed samplers was almost ten times lower. Therefore, retaining the open surface of the sampler in the void was preferred rather than via a protected mesh or perforated barrier. Figure 2 shows the second design, which consisted of a set of 52 mm diameter inert polymer rings that could be used to sandwich the passive sampler. This facilitated more direct exposure to the surrounding air and attachment to surfaces was easily achieved using magnets or hook and loop fasteners (Velcro).

### Trial 3: Explosives vapours in packed shipping containers and vehicles

As a follow on and expansion to Trials 1 and 2, the samplers and their optimised housings were re-trialled in vehicles again (same volume as before) and, also in a large shipping container measuring more than four times the volume of the vehicle and almost double the volume of the room used in Trial 2. Two different explosive sources were used: (a) a Ng-based source which was concealed following its successful detection in Trial 2, and (b) a TNT-based source which was left unconcealed. Samplers were placed at one fixed location in each void (also placed over shipping container air vents internally and externally) over a 72-h period. This trial represented markedly more realistic environments than Trials 1 and 2 as exposure voids were naturally ventilated (unsealed vents) and contained cargo which could have impeded dissipation or acted as a sink for vapours. Furthermore, the minimised number of locations chosen for setting samplers out in each void was performed to facilitate rapid screening, ideally without the requirement to empty the contents of the vehicle/container.

**Figure 3:**
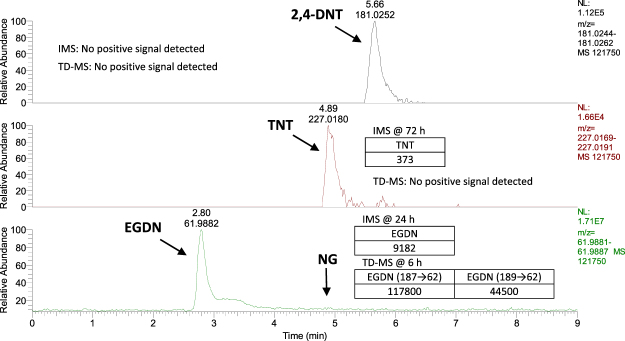
Extracted ion chromatograms (EICs) representing the earliest (6 h) and consistent detection (n = 3) of component residues of TNT flake (1 kg) and Perunit 28 E (1 kg) on passive samplers in a packed shipping container during Trial 3. Unless otherwise stated, the earliest detection time and signal intensity using IMS and TD-MS analysis are given for comparison in inset tables within each EIC. Note: NG and EGDN were both monitored via the nitrate ion EIC (m/z 61.9881–61.9887). Both the explosive sources and passive samplers were deployed at the same time.

**Figure 4:**
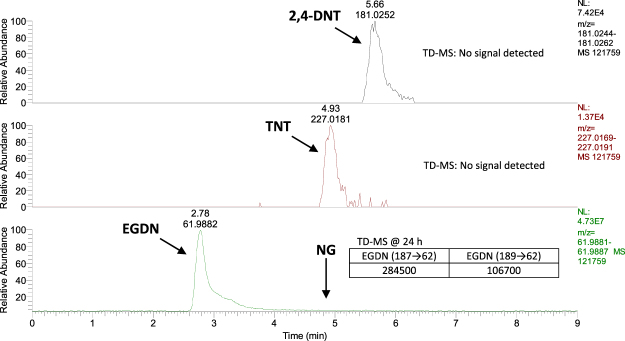
Extracted ion chromatograms (EICs) for component residues consistently detected on passive samplers (n = 3) after 24 h exposure to the exterior vent of the packed shipping container holding TNT flake (1 kg) and Perunit 28 E (1 kg) in Trial 3. Only EGDN was detected on samplers (signal intensities given in inset table) using TD-MS.

As shown in Table 2, EGDN, TNT and 2,4-DNT were detected on passive samplers (n = 3) collected from both the loaded vehicle and shipping container trials at the first 6 h time point using LC-HRMS analysis. When samplers were analysed using TD-MS analysis (n = 3), EGDN was the only analyte detected from 6 h in the container and from 24 h in the van. Using IMS analysis, TNT was detected at 72 h in both spaces and EGDN was detected from 24 h in the container, but later in the van at 72 h, possibly due to its added level of concealment. Figure 3 shows the earliest detection time for each of the deployed explosives using LC-HRMS (6 h for EGDN, TNT and 2,4-DNT), IMS (24 h for EGDN and 72 h for TNT) and TD-MS (6 h for EGDN only) analysis. LC-HRMS enabled the fastest detection of both explosive sources and also detected 2,4-DNT from 6 h which, in contrast, went undetected using IMS and TD-MS analysis. EGDN, TNT and 2,4-DNT were detected from 24 h on samplers placed both inside and outside of all four container vents (24 h was the first sampling time point for samplers at vents) using LC-HRMS only. Figure 4 shows the extracted ion chromatograms (EICs) for explosive components detected on samplers located on the external vents of the shipping container at 24 h. EGDN was the only compound detected on these samplers when analysed by TD-MS, also shown in Fig. 4. Interestingly, samplers located on the outside of the vents were exposed to rain at the end of Day 1 (at ≈6 h). However, sampler performance seemed unaffected by this. Samplers were also exposed to varying temperatures and humidities within the container (see Figure S2 in the supplementary information). However, these fluctuating conditions also did not seem to have any negative impact on sampler performance. EGDN was detected on two samplers located in the driver area of the van after 72 h exposure to 1 kg Perunit 28E, despite the sealing of all vents in the cabin section prior to exposure.

**Table 2:** Detection of commercial and military blasting explosives in Trial 3 for a cargo-loaded van and a packed shipping container and both analysed using LC-UV, TD-MS and IMS.

Void type (volume)	Sampler location	Explosive type (weight)	Exposure time (h)	Analyte detection method and time		
				*LC-HRMS*	*TDMS*	*IMS*
Vehicle (9,000 dm^3^ Transit van packed with furniture)	Inside rear door, approx. 4 m from source (n = 18 prototypes and n = 9 unhoused samplers)	Perunit 28E (1 kg)	72	EGDN ≥ 6 h	EGDN ≥ 24 h	EGDN ≥ 72 h
		TNT (1 kg)		TNT ≥ 6 h 2,4-DNT ≥ 6 h	n.d.	TNT ≥ 72 h
ISO Shipping Container (38,500 dm^3^ semi-filled with boxes)	On interior side of door, source placed in centre of container (n = 18 prototypes and n = 9 unhoused samplers)	Perunit 28E (1 kg)	72	EGDN ≥ 6 h	EGDN ≥ 6 h	EGDN ≥ 24 h
		TNT (1 kg)		TNT ≥ 6 h 2,4-DNT ≥ 6 h	n.d.	TNT ≥ 72 h
	Over vents (n = 4 inside and n = 4 outside)	Perunit 28E (1 kg)	72	EGDN ≥ 24 h	EGDN ≥ 24 h	Not utilised
		TNT (1 kg)		TNT ≥ 24 h 2,4-DNT ≥ 24 h	n.d.	Not utilised

As both the samplers and source were deployed simultaneously, this period included the time required for the vapours to dissipate inside each space (i.e. the gas phase was not yet at equilibrium at the start of the exposure). In the original kinetics experiments detailed in Part A of this paper, 15 µg of each explosive were exposed individually to the sampler over a one week period in glass flasks. The rate of uptake calculated was shown to correlate with vapour pressure (*R*^2^ = 0.6417) across the range of selected explosive types (refer to Part A: Fig. 4). Comparing the volume and the amount of explosive used in the Duran glass bottles (0.135 dm^3^) to those in the van (9000 dm^3^) and container (38,500 dm^3^), the ratio of explosive (weight) to void volume was over 1000-fold and 230-fold lower in the van and container, respectively, compared to the glass bottles (not taking into account the purity of the bulk explosive material). The distance between the source and the samplers placed in the van and container were at least 20-fold further than the distance travelled by vapours produced in the flasks (~11.5 cm). As these simulated operational spaces were also semi-filled, this further reduced the internal volume, but it also provided various surfaces and materials on to which explosive components could undergo absorption, adsorption or transformation. Furthermore, as the van was not hermetically sealed, this may have led to localised air movement. A gap in knowledge exists on the passive dissipation of explosives vapours over time within various void sizes. Of the few reported studies, the majority are based on the development of stand-off spectroscopic systems such as Fourier-transform infrared spectroscopy (FT-IR) for the detection of vapours from explosive sources (mostly in the form of a crystalline solid on a surface) measured at various temperatures and distances^22–24^. Further research on explosive vapour dispersion patterns is therefore required as each vapour may act differently in various environments and these findings would inform where best to place explosives detection systems in the field.

### Trial 4: False alarm trials in semi-operational environments

**Figure 5:**
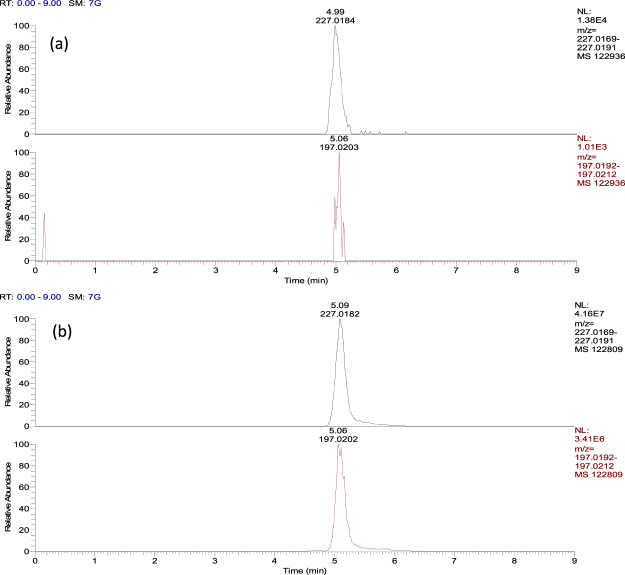
(**a**) Extracted ion chromatograms (EICs) for two TNT ions present on a deployed sampler from the Main Hall 2 location in a public building in Trial 4 (later found to be a nuisance positive indication) and (**b**) for comparison, the same EICs present in a spiked passive sampler extract (5 µg TNT exposed to the sampler in a 0.135 dm^3^ flask over 5 days.

This trial demonstrated the application of the optimised sampler prototype in large, uncontrolled and operationally relevant environments over a 7-d period. No false positive results were recorded for samplers deployed either at the university building or theatre using LC-HRMS or TD-MS analysis together. It should be noted, however, that samplers collected from two separate locations (a dressing room and at the stage door entrance) and analysed by TD-MS yielded one DNT transition i.e. 181 → 135, just above TD-MS threshold levels (5000) at ~7200 counts. Analyte thresholds have been thoroughly investigated by police for application to routine search operations and takes into account common interferences for the selected DNT channels when carrying out thermal desorption of particulate swab samples. However, two transitions are normally required to confirm a positive detection and this was therefore not considered a false positive indication. LC-HRMS analysis of a sampler collected from a location within the public building confirmed detection of TNT on samplers using retention time and two exact m/z ions (Fig. 5(a)). The detection threshold was set at a minimum signal intensity of 2000 counts for all ions without background noise in negative mode using LC-HRMS. HRMS performed at this resolution often yields little/no consistent noise and therefore this threshold was set to avoid electronic noise spikes, which occurred intermittently on this instrument. They did not increase beyond the 2000 count value and had fewer than ten consecutive data-points. An extract of pre-exposed sampler (5 µg TNT over 5 days) is shown in Fig. 5(b) for comparison. The continuous detection of signal across the observed m/z peaks for TNT in Fig. 5 was also confirmed by plotting the intensity peaks in stick view. As samplers were exposed at each location in duplicate, the second sampler had undergone TD-MS analysis and no traces were detected.

According to Crawford and Hill (2013), a low nuisance positive rate is unlikely to be entirely removed due to complex mixtures found in commonly used products. MS and IMS alone cannot provide 100% assurance from nuisance positive results^5^. These analytical techniques are generally used in combination operationally to reduce nuisance positive responses however they cannot be completely eliminated. The source of TNT giving rise to this nuisance positive result is unknown, but not implausible given the consistent presence of explosive screening equipment and personnel, and the high sensitivity of the developed LC-HRMS method for nitroaromatic compounds. This could also represent a legacy trace from a previous training exercise. All attempts to eliminate contamination were followed including use of appropriate PPE and correct sampling and preparation procedures. Therefore, out of n = 72 samplers deployed across all three sites, the false positive rate was <1.4%, which was considered excellent given that they were in place and left unattended for a week in a range of realistic building environments.

### Trial 5: Alternative applications

The flexibility of the sampler for alternative applications was the focus of this trial and was therefore almost entirely different to Trials 1–4. Two applications were tested: (a) its efficiency as a contact swab for explosives particle sampling and (b) as a passive sampler in aquatic media.

#### Contact sampling of particulate explosives on surfaces

This application focussed on five explosives including three with lower vapour pressures than those studied previously (HMX, RDX and pentaerythritol tetranitrate (PETN)). On glass slides (see Table 3), the sorbent film-coated sampler effectively recovered: (a) more PETN residue than the other swab types tested; (b) more TNT residue than Nomex-type swabs; and (c) less 2,4-DNT residue than cotton swabs (though recovery was poor generally across swabs), for n = 20 replicates of each swab.

**Table 3:** Recovery (%) of explosive analytes from swabbing spiked surfaces (15 µg of each analyte) and analysis using LC-UV in Trial 5.

Surface	Swab-type	Recovery ± RSD (%)					
		HMX	RDX	NG	2,4-DNT	PETN	TNT
Glass *n = *20	Cotton	24 ± 6	29 ± 6	13 ± 4	12 ± 4^*^	19 ± 5	17 ± 4
	Nomex	28 ± 5	31 ± 4	12 ± 3	9 ± 2	20 ± 4	15 ± 3
	Sorbent-coated	27 ± 5	32 ± 4	15 ± 4	9 ± 3	28 ± 6^*^	19 ± 5^*^
Plastic *n* = 6	Cotton	21 ± 5^*^	20 ± 4^*^	10 ± 1	7 ± 2	19 ± 2^*^	12 ± 2^*^
	Nomex	18 ± 4	16 ± 3	9 ± 0	5 ± 2	14 ± 4	9 ± 2
	Sorbent-coated	15 ± 2	13 ± 2	10 ± 0	2 ± 2^*^	11 ± 2	7 ± 1
Wood *n* = 6	Cotton	20 ± 10	14 ± 6	16 ± 5	6 ± 2^*^	8 ± 3	6 ± 3
	Nomex	13 ± 5	9 ± 3	12 ± 4	2 ± 2	3 ± 2	3 ± 1
	Sorbent-coated	20 ± 12	14 ± 9	18 ± 7	3 ± 2	6 ± 5	5 ± 3
Metal *n* = 6	Cotton	54 ± 11	56 ± 9	47 ± 8^*^	37 ± 5^*^	53 ± 8	41 ± 5
	Nomex	63 ± 13	56 ± 13	32 ± 10	27 ± 7	48 ± 12	33 ± 8
	Sorbent-coated	34 ± 8^*^	32 ± 9^*^	37 ± 9	13 ± 4	29 ± 8^*^	17 ± 5^*^

^*^Indicates a significant difference (using one-way analysis of variance (ANOVA) with a Tukey’s post-hoc test, *p* < 0.05).

Statistical testing revealed no significant difference was found between the standard deviations yielded for n = 6 or n = 20 replicates across all swab types. Swab efficiency was then compared on plastic, wood and metal surfaces for n = 6 replicates (Table 3). Again, poor recovery of 2,4-DNT was observed, but this is potentially expected given its higher vapour pressure at room temperature in comparison to the other analytes tested. Lower recoveries overall were observed for plastic surfaces than glass across all swab types which may be due to the semi-porous nature of plastic and potential for electrostatic effects. Whilst on average the sorbent film-coated sampler seemed to recover lower amounts of explosives residues from plastic than the uncoated form, a one-way analysis of variance (ANOVA) with a Tukey’s post-hoc test revealed no significant difference (*p* < 0.05) between the two swab types, except for 2,4-DNT where the uncoated form performed statistically better, even though its recovery still remained low. The overall highest average analyte recoveries were obtained using cotton on metal surfaces but these also had the highest variability. Poorer recoveries using the sorbent-coated sampler (especially in comparison to Nomex) could be partially attributed to the swabbing technique used, as repeated swabs of the surface may have stripped the coating from the swab and, along with it, the collected residue. In contrast, previous studies on particulate collection have shown an increase in explosives collection on swab with increased swipes^25^. However, further investigations showed that recovery using the sorbent film-coated swabs decreased with increasing number of swabbing motions done on a non-porous surface (metal). However, the variability between replicates was much higher when the surface was swabbed only once (single left to right motion). In comparison, the residue recovery was improved when swabbing a semi-porous surface five times. Therefore, if utilising the sorbent film coated sampler for particulate swabbing, it is recommended to swab non-porous surfaces two to three times, whereas porous surfaces can be swabbed up to five times. Negative control surfaces (unspiked) and blank swabs (n = 1, for each) were analysed alongside samples and no contamination or interference was identified.

The detection rate of each swab type used on a spiked surface (n = 6) was also considered using IMS as a qualitative detection technique. Following the swabbing of spiked surfaces, sorbent film-coated swabs triggered more detection alarms than cotton and uncoated Nomex swabs (see Figure S3 in the supplementary information). Unused (blank) swabs were run in between each contaminated swab as a negative control to ensure any alarms were not due to carry over between samples. 2,4-DNT was excluded from this experiment due to lower sensitivities for this compound using IMS analysis. Even though 80 ng was spiked on swabs separately to the analyte mixture, none of these alarmed for Ng when analysed by IMS. As Ng has a higher vapour pressure than the other analytes (6.45 × 10^−7^ atm), rapid evaporation is likely to have occurred during the solvent drying step (~5–10 min).

In a second, simulated operational environment experiment using commercial C4 plastic explosives contaminated on identical improvised units bearing light switches, door handles and metal push plates, the sorbent film-coated swabs performed well and contaminated surfaces were all correctly identified using IMS and LC-UV analysis. In addition, no false or nuisance positives were identified, and the sorbent film-coated swabs did not collect or introduce any further interferences from the surfaces than those already detected in the background swabs. Swabs used to collect C4 from the contaminated boards (for both primary and secondary analysis), and analysed using IMS, triggered an alarm for both HMX and RDX, while those analysed using LC-UV detected RDX only. This can be attributed to the intensity of the RDX peak which potentially triggers the IMS detection channels for both RDX and HMX. These channels are shared as both compounds produce M·NO_2_^−^ and M·Cl^−^ adduct ions when ionised^2,26^. Overall, the sorbent coated swabs could be used for recovery of particulate traces, but were not necessarily the best option in all cases.

#### Extraction of explosive residues from wastewater samples

Monitoring illicit residues in influent wastewater can provide intelligence on clandestine lab activity in specific catchment areas. Illicit drug consumption has been recently monitored across 50 European cities and more recently, explosive residues have also been determined in wastewater influent collected from the UK and Finland^18,19,27^. Three sampling techniques were compared and analyte recoveries are reported in Figure S4 for standards in ultrapure water. The highest recoveries were achieved with sampler agitation on a shaker table at 250 rpm. The lowest recoveries were measured using the sorbent film-coated stirrer-bar which may be attributed to a relatively lower surface area. Compounds with the highest affinity for the sorbent were nitroaromatics and nitrate esters. Interestingly, smaller molecules were recovered best using the immersed sampler, while the larger molecules such as HMX and PETN were not consistently recovered. This may have indicated competitive uptake of analytes onto the sampler or sorption to suspended solids, but these were not investigated further herein. Abiotic conditions were investigated in ultrapure water and analyte recoveries were compared using the agitated sampler extraction method and LC-UV analysis. For pH, the recovery for tetryl significantly increased at pH 2 and perhaps as expected due to its higher stability under acidic conditions, but recoveries of Ng, 3,4-DNT, 2-NT, 2,4-DNT and TNT were significantly lower. Overall, a higher salinity did not improve analyte recovery. For temperature, 22 °C yielded higher recoveries overall than at 15 °C, particularly for DNTs and Ng. A sample volume of 100 mL yielded significantly higher recoveries than those measured for 250 and 500 mL.

The uptake kinetics of the sampler over a 24-h period are shown in Figure S5 in the wastewater matrix at more environmentally relevant concentrations, *i.e*. 250 ng L^−1^. While, cumulatively the highest recoveries were achieved at 16 h exposure time, the variance for each compound was high, particularly for the nitroaromatics (%RSD = 40–170%). The optimised exposure time was 24 h for it to reach steady-state equilibrium which may not offer rapid enough results for initial screening purposes, but would offer a simple and cheap sampling/clean-up method for longer-term monitoring of 24 h composite wastewater and potentially at source, either within the composite sampler itself during operation or in subsequent wastewater sub-samples. Method repeatability was investigated in spiked wastewater samples and recoveries (±SD for n = 6 replicates) were measured for TNT (1 ± <1%), 4-NT (77 ± 15%), 2,4-DNT (39 ± 8%), 3,4-DNT (18 ± 3%) and 2,6-DNT (105 ± 133%). 2,6-DNT recoveries were highly variable with the highest RSD value at 133%, however, the DNT compounds shown in the EICs in Figure S6 (a) and (b) were consistently detected on sampler (n = 6) at intensities >1000. The optimised method was applied to a neat 24 h composite sample of influent wastewater where 2,4-DNT was detected. Using three-point standard addition (*R*^2^ = 0.978), the concentration of 2,4-DNT detected was estimated at ~10 ng L^−1^. However, this value is only semi-quantitative and the sampler should be considered primarily as a simple qualitative tool. Previous work in our laboratory has confirmed the occurrence of 2,4-DNT in influent wastewater at concentrations measured up to 303 ng L^−1^ using validated solid phase extraction-based methods coupled to LC-HRMS^18^. In comparison, this passive sampling method offers a simple, less resource intensive approach that could potentially be performed at site. Further method performance research is required to ascertain detection probability of these low-level compounds in influent wastewater.

## Conclusions

The application of a new passive sampler for explosive vapour capture was trialled in a number of different simulated/operational environments and was readily integrated with existing explosive detection instrumentation. Specifically, samplers exposed to commercially available and military explosives successfully recovered detectable component residues of (a) 0.2 kg of concealed Perunit 28E inside a suitcase placed within a void after 4 h (b) 1 kg of concealed Seguridad in a room within 24 h and in an adjoining room within 7 days (c) concealed Perunit 28E (1 kg) inside a loaded van cabin after 24 h and TNT (1 kg) after 72 h and (d) partially concealed Perunit 28E (1 kg) after 24 h and TNT (1 kg) after 72 h, both inside a large semi-filled ISO shipping container. For the latter scenario, EGDN residues were also detected on samplers positioned outside of the container at vent outlets after 24 h and were detectable even after periods of wet weather during the 72 h exposure. The sensitivity, selectivity and practicality of the sampler device was demonstrated in two semi-operational environments and one high-profile operational building that is searched routinely. Overall, no false positives were recorded and a very low nuisance positive rate of <1.4% was achieved. The main limitation of the developed sampler was for capture of explosives traces with vapour pressures lower than that of TNT. Furthermore, though sampler generalisability for uptake and retention of bulk peroxides was not investigated due to operational restrictions, its performance during laboratory scale trials was excellent. In addition to passive sampling of air, application to particulate contact swabbing for lower vapour pressure compounds and extraction of dissolved residues in aqueous matrices were successfully shown. This highlighted the potential for future applications in a range of qualitative monitoring studies for explosives and potentially other compound classes.

## Methods

### Reagents and materials

All solvents, reagents and materials used are as described in Part A^20^. For semi-operational exposures, commercial and military-grade explosives were used as a source of analyte vapours. Commercial-grade explosives included Perunit 28E (NG and EGDN vapour source) and Seguridad (NG and EGDN vapour source) in the form of sticks, and TNT (TNT and DNT vapour source) was sourced as flake material (Eurenco. Massy, France). For trials at EPC UK Ltd., flake material was recast into disks before use. Military-grade explosives included C4 (RDX vapour source) form and Semtex 1 H (RDX and PETN vapour source) as a solid block. For the alternative sampler applications study, additional standard solutions of hexahydro-1,3,5-trinitroso-1,3,5-triazine (R-salt, >99% purity in acetonitrile), HMX (>99% purity in 50:50 acetonitrile:methanol), RDX (>98% purity in 50:50 acetonitrile:methanol), nitrobenzene (NB, >99% purity in methanol), erythritol tetranitrate (ETN, >99% purity in acetonitrile), tetryl (>98%, purity in 50:50 acetonitrile:methanol) and PETN (>99% purity in methanol) were sourced at 1000 mg L^−1^ (and 100 mg/L for R-Salt only) from Kinesis Ltd. (St. Neots, UK). Acquisition, storage, handling and setting of bulk explosives in all trials was performed by licenced technicians from the Metropolitan Police Service (London, UK), Event Horizon (Somerset, UK) and/or EPC UK Ltd. (Essex, UK) and in compliance with the Explosives Act 1875^28^ and The Explosives Regulations 2014^29^.

### Instrumentation and analytical methods

All analytical methods and conditions applied to the analysis of samplers used in semi-operational environments (IMS, TD-MS and LC-HRMS) are as described in Part A. Alternative sampler applications for explosive uptake in ultrapure water and as a particulate swab used LC-UV performed on an Agilent 1100 Series liquid chromatograph (Agilent Technologies Ltd., Cheshire, UK) using an ACE C_18_ AR column (150 × 2.1 mm, 3 μm, Advanced Chromatography Technologies Ltd., Berkshire, UK). Two different elution methods were used. The first, an isocratic elution method, comprised of an 8-min run time with a 30:70 (*v/v*) 8 mM ammonium acetate in water:methanol mobile phase, an injection volume of 2 μL, a flow rate of 0.2 mL min^−1^ and a column temperature of 45 °C. This was used to assess particulate recovery from surfaces. A second gradient method, for the separation of more compounds and analysis of recovery from aqueous matrices, consisted of a 75 min run with a 90:10 (*v/v*) 8 mM ammonium acetate in water:methanol mobile phase (A) and a 10:90 (*v/v*) 8 mM ammonium acetate in water:methanol mobile phase (B). The gradient consisted of a ramp from 40% to 100% B for 30 min, an isocratic step at 100% B from 30 to 40.5 min and then an isocratic step at 40% B from 40.5 to 75 min. The flow rate was 0.15 mL min^−1^ and the column temperature was 20 °C with an injection volume of 1 μL. For wastewater analysis, a recently developed and validated LC-HRMS method was applied as described by Rapp-Wright *et al*.^18^.

### Trial 1: Preliminary trials in baggage and vehicles

This trial was conducted at EPC-UK, Essex, UK from 18^th^–21^st^ August, 2014 (weather data given in Table S1 in the supplemental information). The first experiment involved detection of vapours in baggage. Perunit 28E (0.2 kg), TNT (0.6 kg) and Semtex 1 H (3 kg) were individually wrapped in a cotton towel, placed in separate sealed suitcases (20 dm^3^) and individually placed inside a sealed plastic container (64 dm^3^). Plastic containers were stored in a closed polyester fabric tent during the day and secured indoors overnight (to comply with standard safety protocols at the trial site). Samplers were placed (a) inside the plastic container, but outside of the suitcase (n = 6) and collected at 4, 23, 29, 47 h, (b) inside the suitcase (n = 3) and collected at 47 h, and (c) from inside the tent (n = 3) collected at 47 h. A plastic container with no explosive (negative control) was also placed in the tent and run in parallel, but with samplers collected at 47 h only (n = 3). Samplers were also placed inside the tent, but externally to the plastic containers to determine any potential contamination.

The second experiment involved commercial and military-grade explosives inside the cabins of two large transit vans set down within a secure holding site. Prior to exposure, all vents were covered with masking tape and the inside of the cabins wrapped with brown paper to minimise soaking of explosives vapours into the van interior. All vans were tested for the presence of explosive residues beforehand and were negative. Samplers were hung from a plastic household 20-peg airer attached to the back door of each cabin to enable consistent replication. The explosive sources were placed in an open container at the opposite end of the van. Samplers were exposed to Perunit 28E (0.4 kg) in Van 1 for a 23 h period and collected after 4 and 23 h (n = 6 per time point). Following this experiment, the van was aerated (and tested negative using IMS) and fresh samplers were exposed the next day to ten-fold the amount of Perunit 28E (4 kg) in the same van and collected at 4 and 22 h (n = 6 per time point). For the larger Perunit 28E exposure, samplers were also placed outside the van from 4 h after the initial exposure and collected after 18 h. This included samplers under the chassis (n = 6), the back door (n = 1) and on the side of van (n = 3). A third exposure was performed in Van 2 using TNT (3 kg) and samplers were collected at 4, 23, 30, 47 h (n = 6 per time point). For all three exposures, samplers (n = 3) were also placed in the driver’s cabin and collected at the final time point for each exposure. Prior to all exposures, a control experiment was carried out in the van and samplers were exposed and collected at 24 h (n = 6). All samplers were analysed directly by LC-UV, IMS and TDMS.

### Trial 2: Room-scale exposures and determination of optimal sampler range

A second trial was run at a secure Metropolitan Police facility within its Greater London jurisdiction from 10^th^–24^th^ November 2015 (performed at room temperature at ~17 °C). This trial was designed to assess and determine the effective sampler distance range and the optimum sampler location, *i.e*. height and distance from source, within a room containing the explosive, and the potential for detection in a connecting room (no source present). All vents in Rooms A and B were covered with masking tape and surfaces in Room A were covered with plastic to minimise soakage of explosives vapours. Rooms A and B were separated by a locked door that was kept closed (but not sealed) during the controls and exposures, except to allow periodic collection of samplers as required. Background testing was performed by suspending samplers in Rooms A and B at mid-height for 7 days prior to exposure. Following collection of these controls, fresh samplers were deployed at several distances from the source within Room A (0.9–3.3 m) and Room B (3–10 m) and at three heights (low, middle and high) (see Figures S1 in the supplementary information). The source used was an Ng-based commercial explosive (1.2 kg Seguridad) which was placed inside a locked drawer of a cabinet marked at Location X in Room A. Samplers (n = 3) were exposed and collected after 2 and 6 h (from all locations in Room A and above the door in Room B only) and 24, 48, 72, 168 h (from all locations). Samplers collected at different time points and locations were placed in separate zip-sealed plastic bags for storage until analysis. Samplers were then transported to the laboratory, extracted and analysed within 48 hours using the optimised LC-UV method described in Part A.

### Trial 3: Explosives vapours in packed shipping containers and vehicles

The final trial was conducted at Event Horizon, Somerset, UK from 24^th^–28^th^ July 2016 (weather data in Table S1 in the supplemental information). Two exposures were performed over 72 h and samplers (n = 9) were collected at 6, 24 and 72 h. The first time point at 6 h was selected as it fit well within an 8-h working day. Housed sampler prototypes were collected for IMS and TD-MS analysis (n = 3 for each) and unhoused samplers (n = 3) were used for LC-HRMS analysis only, for quantitative comparison. Sampler prototypes (shown in Fig. 2) consisted of an inert polymer support ring within which the sampler was clamped and could be mounted on to a surface using either Velcro or magnets (see S1.0 in the supplemental information for more detail on the optimisation of the sampler housing).

Background (negative control) measurements of a large, new metal ISO shipping container (38,500 dm^3^) were made following exposure of samplers (n = 9) within the empty container for ≈60 h. This trial also included deployment of the optimised housing design for the passive samplers to enable the most effective vapour uptake. The container was then half-filled with large empty cardboard boxes wrapped in cling film to simulate cargo. Housed sampler prototypes were attached magnetically to the inside of the primary container door (n = 18). A second set of unhoused samplers (i.e. the bare sampler) were deployed inside the door of the container via a peg airer (n = 18). Samplers (n = 8) were also placed over the four vents located in the four upper corners of the container (both internally and externally to the container) and sampled at 24 and 72 h for LC-HRMS and TD-MS analysis. Two sources of explosive were used together in this trial, *i.e*. TNT flake (1 kg) placed in an open tray on top of a cling-filmed cardboard box in the centre of the shipping container (mid-height) and Perunit 28E (1 kg) concealed in a nylon bag and placed in a plastic crate with air holes (low height).

A large van (9,000 dm^3^ internal volume) was utilised again in a second experiment to detect concealed explosives within a cargo load. As in previous vehicle exposures, all vents were covered with masking tape and the inside of the cabin covered with brown paper. The van was then packed with a two-seater sofa and a varnished wooden cabinet to provide obstruction and potential vapour sink. As a negative control measurement, samplers (n = 9) were exposed to the packed van environment for ≈20 h before entering the holding environment. Once on site, optimised sampler prototypes were attached with magnets to the inside of the van ceiling and another set of unhoused samplers were hung just inside the back door of the van for comparison. Unhoused samplers (n = 6) were also placed on the dashboard in the front cabin. Two sources were also used in this set-up, i.e. TNT flake (1 kg) placed in an open container and left on top of the cabinet, and Perunit 28E (1 kg) concealed in a nylon bag and placed in a closed drawer in the cabinet.

### Trial 4: False alarm trials in semi-operational environments

Sampler prototypes were deployed for a period of one week at 12 different locations within each of (a) a University; (b) a large theatre and; (c) a public building regarded as critical national infrastructure. All venues were located in Central London, publicly accessible and represented typically searched or potentially targeted buildings and areas. Sampler prototypes were deployed in duplicate to enable both TD-MS (n = 1) and LC-HRMS (n = 1) analysis at each location. Sampler prototype deployment locations included hallways, stairwells, cloakrooms, classrooms, offices, conference rooms and dressing rooms (more specific room details are given in S2.0 of the supplemental information). A positive identification was recorded when the results obtained from any one sampler, despite the analytical method used, indicated the presence of a target residue above the limit of detection. Any confirmed positive results obtained were counted and grouped. A nuisance positive rate was calculated as a percentage of the total number of samplers deployed across all venues (n = 72). Nuisance positives are defined here as those analytical results which confirmed the presence of an explosives-related compound on a sampler, but where no device representing an imminent threat was discovered at the venue following a subsequent thorough search.

### Trial 5: Alternative applications

#### Contact sampling of particulate explosives on surfaces

Three types of swabbing material were considered for comparison of (a) particulate explosives recovery and (b) detection probability. These were cotton, Nomex and the sorbent film-coated samplers. Selected surfaces including glass (glass microscope slides, Thermo Scientific, UK), plastic acetate (overhead projector sheets, Office Depot, UK), laminated wood (Ikea wooden shelf, UK) and metal (Steel push plate, Bulk Hardware Ltd. UK) were spiked with 80 ng (for IMS analysis) and 15 µg (for LC-UV analysis) of each analyte (HMX, RDX, ng, 2,4-DNT, TNT, and PETN) and allowed to air dry. For all surfaces, spiking was performed for n = 6 replicates for each swab type and analytical method combination (*i.e*. n = 6 replicates of four surfaces at two spiking levels and 3 swab types = 144 replicates in total). Percentage recovery (quantitative) was determined using LC-UV and detection probability (qualitative) was determined using IMS. In particular, percentage recovery from glass (measured using LC-UV only) was studied in more depth for n = 20 replicates for each swab type (total n = 60 extra replicates). This was performed to test whether there was any added value by including more replicates in the determination of recovery given the potential for high variance involved. Swabs were mounted in sampling wand normally used to collect particulate traces prior to analysing with IMS detectors (Smiths Detection, London, UK) and surfaces were swabbed back and forth five times. Detection probability was defined as the number of times the instrument successfully detected the analytes in a mixture (except for Ng which was not detected in the analyte mix, potentially due to ion interference and/or its higher vapour pressure, and was swabbed separately) over n = 6 replicates for all surface types. The performance of the sorbent film-coated swab in a simulated operational environment was also investigated by conducting a blind test. Fourteen wooden boards were constructed with a door handle (either brass or steel), a plastic light switch and an additional wooden board and were divided into two groups (A and B, n = 7 for each group). A single surface on one board per group was contaminated via secondary transfer of fingerprints spiked with either C4 or Semtex 1 H. The identity of the contaminated board was unknown to the analyst. Prior to spiking, all boards were cleaned using Isopropanol SF Isoclene Cleaning Spray (AF International, Leicestershire, UK) and was confirmed to be contamination-free using IMS analysis. For Group A, swabs of each surface on individual boards were analysed using LC-UV (primary analysis). The boards were then subsequently and sequentially re-swabbed and analysed with IMS (secondary analysis). For Group B, the opposite order of analytical testing was conducted, *i.e*. swabs were analysed using IMS, then re-swabbed and analysed using LC-UV. As demonstrated by Song-Im (2012), dry swabs such as cotton do not have a 100% collection efficiency for organic explosives on a range of materials, therefore, this secondary sampling approach could be performed to confirm the primary analysis and to potentially help further identify any false positives^30^.

#### Extraction and concentration of trace explosives from raw wastewater samples

The potential for application of the developed passive samplers as an alternative to solid phase extraction for wastewater analysis was investigated. Raw wastewater grab samples were collected after the primary clarifier of a major Central London treatment works over a four day period mid-October 2015. A single 24 h composite influent wastewater sample was collected from the same location during mid-March 2015. Samples were transported to the laboratory in 1 L bottles and transferred into 500 mL Nalgene bottles for storage at −20 °C. Defrosted samples were filtered through a GF/F glass microfiber filter (Whatman, Buckinghamshire, UK) under vacuum and combined in equal volumes to produce a pooled sample. Recovery assessment (n = 6) was initially performed in deionised water (100 mL) spiked with R-Salt, HMX, RDX, NB, ng, 3,4-DNT, 2-NT, 3-NT, 4-NT, 2,6-DNT, 2,4-DNT, ETN, TNT, PETN and tetryl at 25 ng mL^−1^ and EGDN at 12.5 ng mL^−1^. Spiked water samples were extracted over a relatively long 24 h period and analysed using the gradient LC-UV method described above. The main envisaged future application of this approach was as a convenient way to extract and concentrate trace explosives in the on-board composite sampler collection vessel at source rather than at the laboratory for rapid qualitative screening of wastewater as an early warning mechanism for clandestine manufacturing activity. Three different modes of uptake were investigated in deionised water, *i.e*. static uptake where a sampler was placed in the aqueous sample without agitation; and two modes of dynamic uptake where a sampler was placed in the aqueous sample and agitated on a shaker table at 250 rpm and where a sorbent film-coated magnetic stir-bar (35.5 × 6.5 mm, Sigma-Aldrich, UK) was placed in the aqueous sample and agitated at 350 rpm. Various abiotic conditions were also optimised including pH (2 and 7), salinity (0, 2 and 10%), temperature (15 and 22 °C), and sample volume (100, 250 and 500 mL) for n = 3 replicates each. The exposure time was optimised in wastewater samples spiked at 0.25 ng mL^−1^ (EGDN spiked at 0.125 ng mL^−1^). The uptake kinetics of the selected passive sampling mode was investigated over the 24 h period at intervals of 2, 4, 8, 16, 24 h (n = 3 for each time point). The optimised method (pH 7, 0% salinity, 22 °C, 100 mL sample volume, 24 h exposure time) was then applied to a 24 h composite influent sample and detected explosive components were then semi-quantified using standard addition calibration (0, 100, 250, 500 ng L^−1^ spiked samples).

## Electronic supplementary material

Supplementary Information
