# Supplementary material for: Sorbent Film-Coated Passive Samplers for Explosives Vapour Detection Part B: Deployment in Semi-Operational Environments and Alternative Applications
Source: Sci Rep. 2018 Apr 11;8:5816. doi: 10.1038/s41598-018-24245-x (PMC5895787; doi:10.1038/s41598-018-24245-x)
Supplement: Supplementary file 1 — Supplementary Information [file 41598_2018_24245_MOESM1_ESM.pdf]

ELECTRONIC SUPPLEMENTARY INFORMATION

SORBENT FILM-COATED PASSIVE SAMPLERS FOR EXPLOSIVES VAPOUR DETECTION PART B: DEPLOYMENT IN SEMI-OPERATIONAL ENVIRONMENTS AND ALTERNATIVE APPLICATIONS

Gillian L. McEneff[a*], Alexandra Richardson[a], Tony Webb[b], Dan Wood[b], Bronagh Murphy[a], Rachel Irlam[a], Jim Mills[c], David Green[a], Leon P. Barron[a]

[a]*King's Forensics, School of Population Health & Environmental Sciences, Faculty of Life Sciences & Medicine, King's College London, SE1 9NH, London, United Kingdom.*
[b]*Threat Mitigation Technologies, Metropolitan Police Service, Denmark Hill Wireless Station, 113 Grove Park, London, SE5 8LE, United Kingdom.*
[c]*Air Monitors Ltd., 2/3 Miller Court, Severn Drive, Tewkesbury, Gloucestershire, GL20 8DN, United Kingdom.*

Table of contents:

S1.0 Optimisation of sampler housing S3
S2.0 Sampler deployment locations at sites used in Trial 4 .... S3

List of tables:

Table S1. Temperature (°C) and sunshine (hours) recorded at neighboring weather stations over trial periods S4

List of figures:

Figure S1. Sampler set-up in and around Rooms A and B (Trial 2). Location and heights of samplers are marked on the legend with the hide (explosive source) marked as x........ S5
Figure S2. Temperature and humidity recorded in container (Trial 3) over 72 h exposure period S5
Figure S3. Detection rate of organic explosives on three different swabs, cotton (grey bars), Nomex (white bars) and sorbent film-coated swabs (black bars), on various surfaces using IMS analysis........ S6
Figure S4. Comparison of the three sampling techniques in relation to recovery of analytes from deionised water samples (100 mL) S6
Figure S5. Uptake kinetics of the optimised sampler method at 250 ng L^{-1} in a spiked wastewater sample using LC-HRMS analysis in Trial 5. .... S7
Figure S6. LC-HRMS extracted ion chromatograms of (a) 2,4-DNT and (b) 3,4-DNT and 2,6-DNT, detected on sampler swabs in spiked (0.25 ng mL-1) influent wastewater (pooled) and (c) 2,4-DNT collected on sampler swabs in neat 24-hr composite influent wastewater during Trial 5. .... S7

S1.0 Optimisation of sampler housing

During Trial 2, a selection of sampler housing designs were exposed over 168 h in close proximity to samplers hanging freely without housings (i.e. via pegs), and took the form of (i) a plug-in casing, placed low to match the general height of plug sockets, and (ii) a smoke alarm casing, which was attached high on a wall using Velcro. Both prototypes were tested with (n=1) and without (n=1) a metallic mesh filter over the front of the sampler. The addition of the mesh protector within the casing was found to yield lower analyte uptake on samplers at a higher height in the smoke alarm casing but showed no difference in the plug prototypes placed at low heights. Although there were several holes drilled into each casing to promote air flow through the holder and over the sampler, it was apparent from the results that the casing acted as a physical barrier between the sampler and the ambient environment. The optimised sampler prototype was utilised during Trials 3 and 4, and yielded similar recoveries to those samplers without packaging.

S2.0 Sampler deployment locations at sites used in Trial 4

Site A: University Campus (n=2 samplers deployed at each location)

a) Classroom 1 (Above door)
b) Classroom 2 (Above door 1)
c) Classroom 2 (Above door 2)
d) Classroom 3 (Above door 1)
e) Classroom 3 (Above door 2)
f) Stairwell on 4th Floor (Ceiling)
g) Office (Beside door)
h) Office (Back wall)
i) End of Corridor B (Above door)
j) End of Corridor C (Above door)
k) End of East Corridor (Beside fire exit stairwell, above door)
l) Middle of East Corridor (Ceiling)

Site B: Theatre (n=2 samplers deployed at each location)

a) Dressing Room 1(Side of fridge closest to door)

b) Dressing Room 2 (Side of fridge)
c) Stage Door Entrance (Front of electric box at door)
d) Stalls Bar (Above bar counter, behind shutter)
e) Box Office (Above door sitting on top of inside ledge)
f) Cloakroom in the Foyer (On metal casing on inside wall, right side of door)
g) Upper Circle Auditorium (Right spotlight)
h) Office (Front of electric box on left-side wall)
i) Dress Circle Bar (Side of fridge)
j) Upper Circle Bar (Side of fridge at far end of bar)
k) Crew Room (On metal casing on ceiling, mid room)
l) Orchestra Room (On electric box against back wall)

Site C: Public Building (n=2 samplers deployed at each location)
a) Two floors above Main Hall 1 (On metal framework above)
b) Above Main Hall 1 (On metal framework above)
c) Below Main Hall 1 (On metal framework above)
d) In Main Hall (Void within chair 1)
e) In Main Hall (Void within chair 2)
f) Suite (On top of wooden dresser)
g) Meeting Room (On top of CCTV power supply)
h) Sub-Basement (Below Main hall 2)
i) Basement (Below Main hall 2)
j) Above Main Hall 2 (On metal framework above)
k) In Main Hall 2 (Void within central table)*
l) Adjacent Room to Main Hall 2 (On top of wooden dresser)

*one sampler at this location tested positive for TNT using LC-HRMS analysis

Table S1. Temperature (°C) and sunshine (hours) recorded at neighbouring weather stations over trial periods.

Trial (Weather station location)	Date		Temperature (°C)		Sunshine (Hours)
			Min.	Max.	
Trial 1 (Wattisham)	August 2014	18th	10	19	4
		19th	8	16	8
		20th	6	18	6
		21st	5	19	8
Trial 3 (Yeovil)	July 2014	24th	13	20	1
		25th	13	21	5
		26th	11	20	3
		27th	16	23	2
		28th	10	24	4

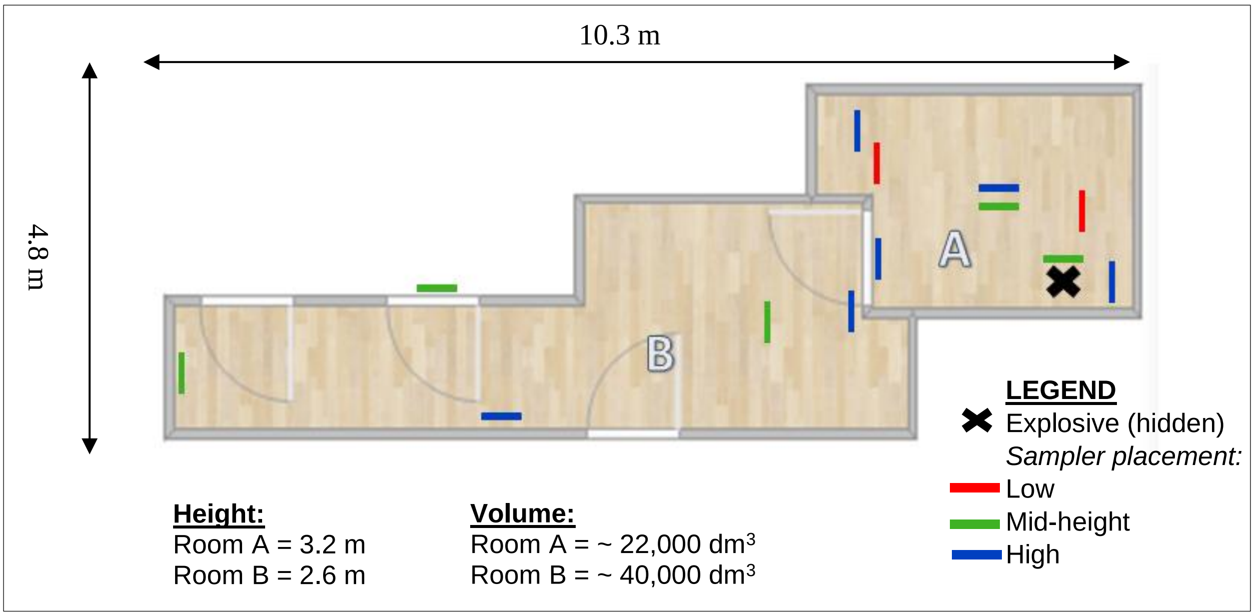

Figure S1. Passive sampler set-up across Rooms A and B (Trial 2). Location and heights of samplers are marked in the legend with the explosive source marked as ‘X’ (which was hidden inside a small locked cabinet).

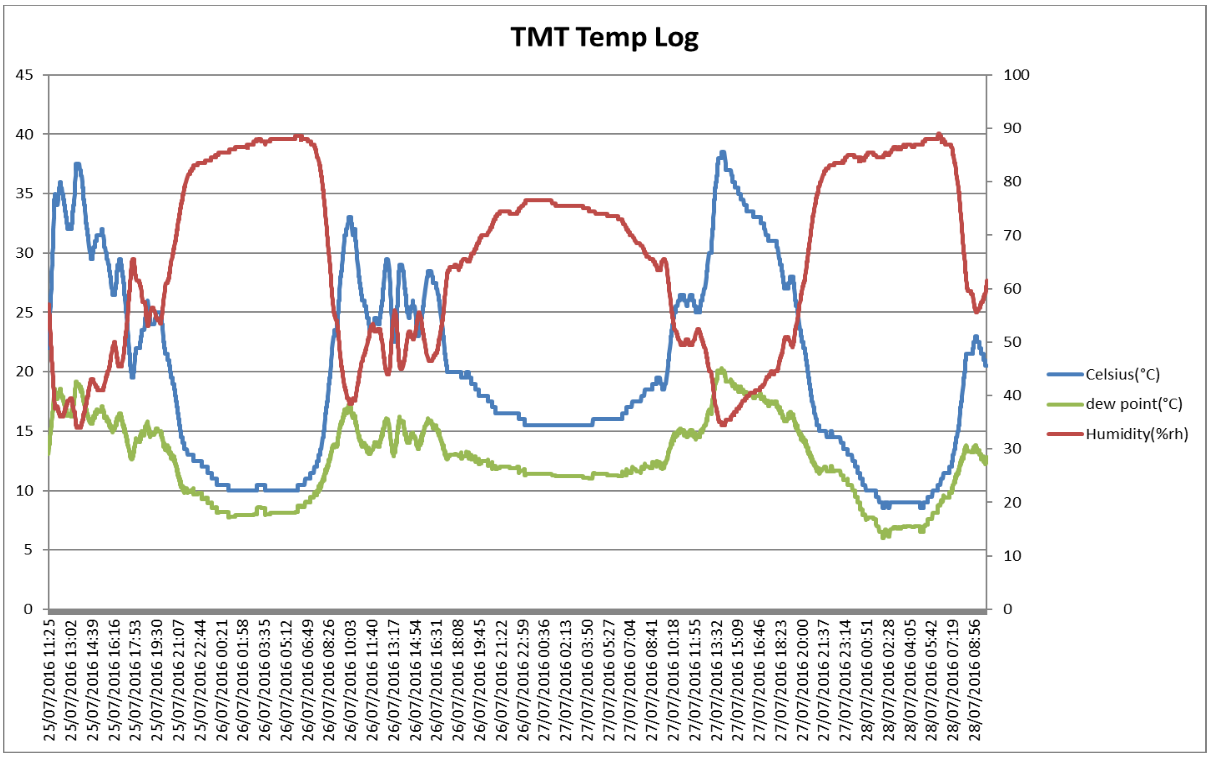

Figure S2. Temperature and humidity recorded in container (Trial 3) over 72 h exposure period.

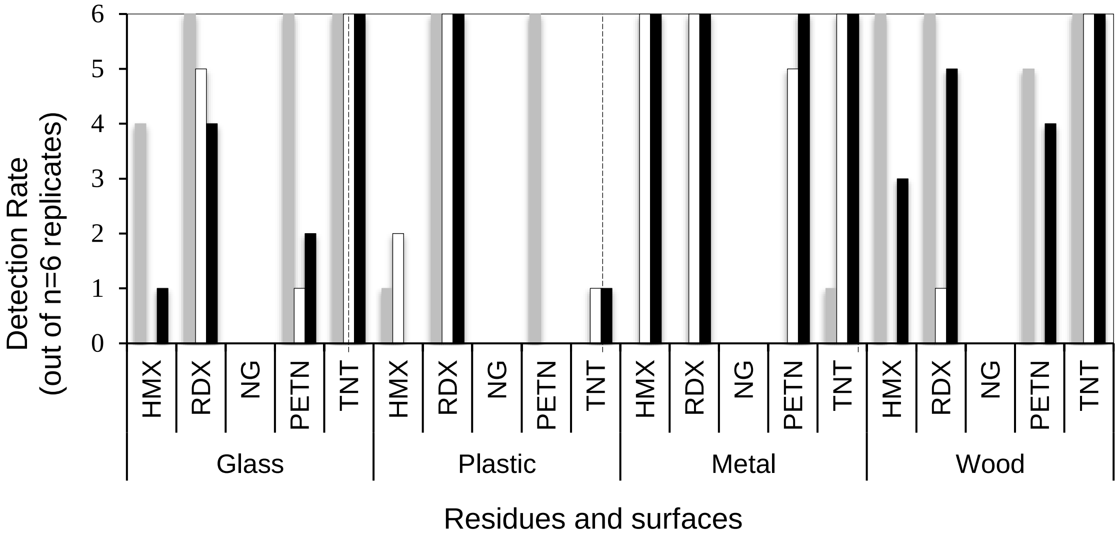

Figure S3. Detection rate of organic explosives on three different swabs, cotton (grey bars), Nomex (white bars) and sorbent film-coated swabs (black bars), on various surfaces using IMS analysis.

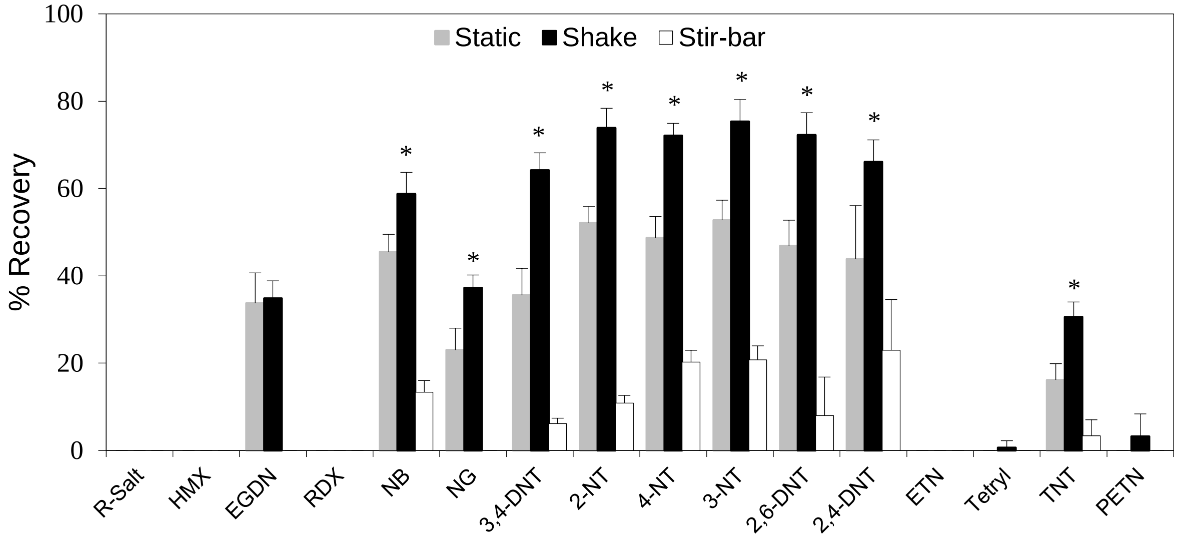

Figure S4. Comparison of the three sampling techniques in relation to recovery of analytes from deionised water samples (100 mL). Error bars represent the relative standard deviation for n=6 replicates, significant differences are indicated.

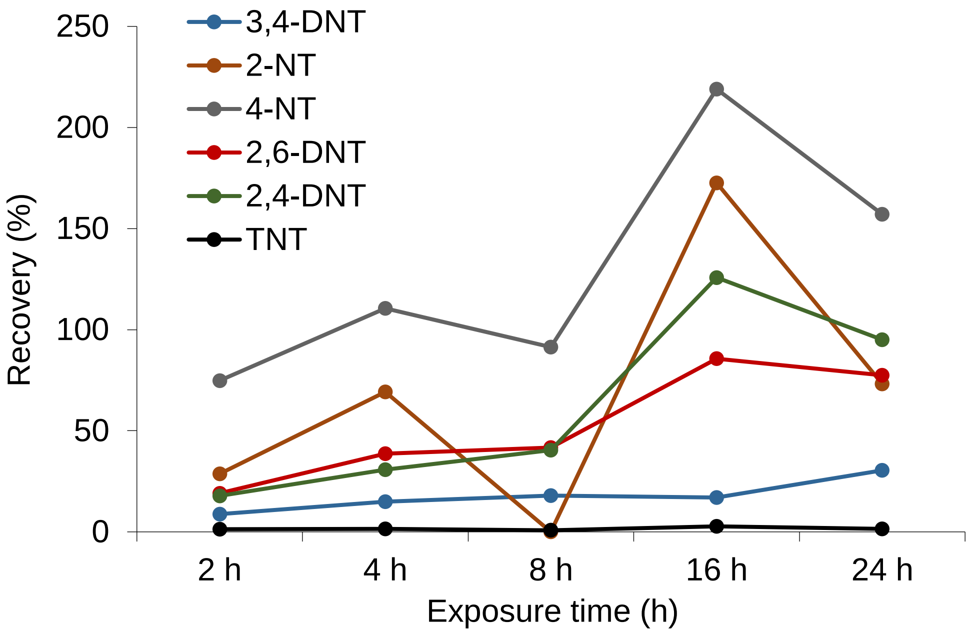

Figure S5. Uptake kinetics of the optimised sampler method at 250 ng L^{-1} in a spiked wastewater sample using LC-HRMS analysis in Trial 5.

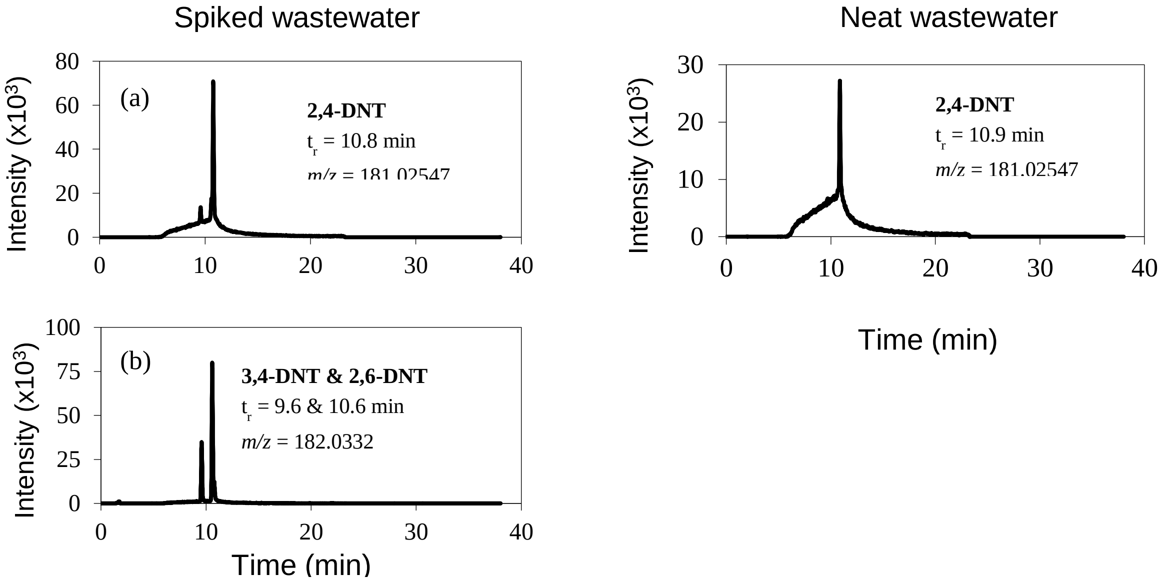

Figure S6. LC-HRMS extracted ion chromatograms of (a) 2,4-DNT and (b) 3,4-DNT and 2,6-DNT, detected on sampler swabs in spiked (0.25 ng mL^{-1}) influent wastewater (pooled) and (c) 2,4-DNT collected on sampler swabs in neat 24-hr composite influent wastewater during Trial 5.
